# First-in-human, dose-escalation, phase 1 study of anti-angiopoietin-2 LY3127804 as monotherapy and in combination with ramucirumab in patients with advanced solid tumours

**DOI:** 10.1038/s41416-020-1011-7

**Published:** 2020-08-03

**Authors:** Juan Martin-Liberal, Antoine Hollebecque, Philippe Aftimos, Christiane Jungels, Patricia Martin-Romano, Jordi Rodon, Jill Dolores Kremer, Wei Zhang, Johanna Bendell

**Affiliations:** 1 Department of Medical Oncology, Vall d’Hebrón Institute of Oncology (VHIO), Barcelona, Spain; 2 Department of Medical Oncology, Catalan Institute of Oncology (ICO), Barcelona, Spain; 3 Department of Adult Medicine, Gustave Roussy, Paris, France; 4 Department of Medical Oncology, Institut Jules Bordet, Université Libre de Bruxelles, Brussels, Belgium; 5 Eli Lilly and Company, Indianapolis, IN USA; 6 Department of Medical Oncology, Sarah Cannon Research Institute, Nashville, TN USA

**Keywords:** Drug development, Drug development

## Abstract

**Background:**

This is the first-in-human study of novel anti-angiopoietin-2 (Ang-2) monoclonal antibody LY3127804 as monotherapy and in combination with ramucirumab in advanced solid tumours.

**Methods:**

Patients received intravenous LY3127804 monotherapy (4, 8, 12, 16, 20 and 27 mg/kg) in part A; LY3127804 (8, 12, 16, 20 and 27 mg/kg) with 8 mg/kg ramucirumab in part B; and LY3127804 (20 mg/kg) with 12 mg/kg ramucirumab in part C. Treatments were administered every 2 weeks (Q2W) during 28-day cycles. Dose-escalation was based on cycle 1 dose-limiting toxicities (DLTs).

**Results:**

Sixty-two patients were treated in part A (*n* = 20), part B (*n* = 35) and part C (*n* = 7). Constipation, diarrhoea and fatigue were the most common treatment-emergent adverse events (TEAEs) in part A; hypertension and peripheral oedema were the most frequent TEAE in parts B and C. No DLT was observed and maximum tolerated dose for LY3127804 was not reached. Four patients achieved partial response with combination therapy (clear cell endometrial carcinoma, cervix squamous cell carcinoma, carcinoma of unknown primary and gastroesophageal junction carcinoma), 29 achieved stable disease, and 24 had progressive disease.

**Conclusions:**

LY3127804 monotherapy and its combination with ramucirumab are well tolerated. LY3127804 20 mg/kg was the recommended Phase 2 dose.

## Background

Angiogenesis is a vital process for growth, proliferation and metastasis of tumours. Multiple vascular factors play an important role in angiogenesis, with vascular endothelial growth factors and receptors (VEGF-VEGFRs) among the most potent angiogenic systems^1^ in promoting the growth of solid organ malignancies.^2^ Therefore, anti-VEGF-VEGFR therapies have become the standards of care (SoC) for several solid malignant conditions including colorectal cancer, renal cancer, hepatocellular cancer and ovarian cancer.^3^

The angiopoietin-Tie receptor axis is another important proangiogenic system that promotes tumour progression. The system comprises the Tie-1 and Tie-2 tyrosine kinase receptors and angiopoietin 1–4 (Ang-1 to Ang4) as ligands to Tie-2.^4^ Ang-1 and Ang-2 are the most extensively studied Ang ligands. While Ang-1 is responsible for vessel maturation, and mediation of endothelial cell migration, adhesion and survival, Ang-2 promotes apoptosis and vascular regression, and causes disruption of the connections between the endothelium and perivascular cells.^4^ Several Ang-2 inhibitors have been evaluated for their safety and efficacy in solid tumours. Phase 1 studies of AMG 386 (trebananib; an Ang-1 and Ang-2 inhibitor) as monotherapy^5^ and in combination with anti-VEGF therapy,^6,7^ showed good tolerance and antitumour activity in patients with advanced solid tumours. Similar activity results were observed with the selective anti-Ang-2 monoclonal antibody (MAb) MEDI3617 combined with bevacizumab, although further development of the drug was discontinued due to toxicity.^8^ Monotherapy with nesvacumab (REGN910), another Ang-2 inhibitor, also showed acceptable safety and antitumour activity in patients with treatment-refractory advanced solid tumours.^9^ In addition, combination of Ang-2 inhibitors and chemotherapy also showed no new safety signals in the recently concluded TRINOVA studies.^10–12^ Simultaneous inhibition of Ang-2/Tie-2 and VEGFA/VEGFR2 pathways may play a role in vessel destabilisation and have an effect on the proliferation and migration of endothelial cells leading to a greater anti-angiogenic effect in the treatment of malignancies. Therefore, a combination of Ang-2 inhibitors and anti-VEGF therapies may be considered promising in patients with multiple tumour types.^13^

LY3127804 (Eli Lilly and Company; Indianapolis, IN, USA) (https://adisinsight.springer.com/drugs/800043698) is a novel anti-Ang-2, humanised immunoglobulin G4 (IgG4) MAb, which is under clinical development for the treatment of advanced/metastatic solid tumours. LY3127804 and aflibercept combination has shown efficacy in mouse models.^14^ Furthermore, LY3127804 in combination with DC101, a murine anti-VEGFR2 antibody, had greater efficacy than LY3127804 monotherapy in multiple patient-derived xenograft models including models for non-small cell lung cancer (NSCLC) and ovarian cancer.^14^ We report the findings of the first-in-human, Phase 1, dose-escalation study JQBA, which assessed the safety, tolerability, pharmacokinetics (PK), and pharmacodynamics (PD) of LY3127804 in patients with advanced solid tumours. In this trial, LY3127804 was administered as monotherapy or in combination with ramucirumab, a MAb targeting VEGFR2.

## Methods

### Study design and patient population

The JQBA study was the first-in-human, non-randomised, open-label, dose-escalation, Phase 1 study of LY3127804 in patients with advanced/metastatic solid tumours who had disease progression with SoC or for whom there was no SoC treatment available. Patients from four centres in Belgium, France, Spain and the United States were enrolled between November 19, 2015 and November 23, 2017.

Institutional review boards of each centre approved the study protocol before initiation of patient enrolment. The study adhered to the Declaration of Helsinki, the International Conference on Harmonization Guidelines for Good Clinical Practice, Council for International Organizations of Medical Sciences and applicable local regulations. All patients provided informed consent before enrolment. The study was registered in ClinicalTrials.gov (NCT02597036).^15^

The study included adult patients with histologically or cytologically confirmed advanced/metastatic solid tumours who were appropriate candidates for experimental therapy in the absence of a standard therapy or for whom available standard therapies had failed to provide clinical benefit for their disease, who refused standard therapies, or who were not a candidate for those therapies, and had progressive disease prior to study entry. Study patients were required to have measurable or evaluable disease according to Response Evaluation Criteria in Solid Tumors (RECIST) version 1.1;^16^ life expectancy that would allow completion of at least two treatment cycles; adequate hematologic, hepatic, renal, and coagulation functions; Eastern Cooperative Oncology Group performance status of ≤1; to have discontinued receiving any anticancer therapy for the last 28 days or five half-lives of the previous treatment before enrolment, whichever was shorter, and recovered from the acute effects of therapy.

Patients were excluded if they had any contraindication to LY3127804 or other MAbs, were previously treated with Ang-2 inhibitors, had any second primary active malignancy, or had any bleeding disorder or thromboembolic event. Patients with active/known fungal/bacterial/viral infection, history of major surgery within 28 days prior to randomisation, or placement of a central venous access device within 7 days before receiving treatment, or any planned major surgery during the trial, and patients (with non-small cell lung cancer only) with gross haemoptysis or major blood vessel encasement were also excluded.

### Treatment plan

The study was conducted in three parts (Supplementary Fig. S1). Maximum duration of treatment was not predetermined. In part A, dose-escalation followed a 3 + 3 study design and the patients with any solid malignancy were treated in six cohorts (Cohort A1–A6). Cohorts A1–A6 received LY3127804 monotherapy doses of 4, 8, 12, 16, 20 and 27 mg/kg, respectively, intravenously (IV) once every 2 weeks (Q2W). In part B, patients were treated in five cohorts (Cohort B2-B6) with LY3127804 doses of 8, 12, 16, 20 and 27 mg/kg, respectively, IV Q2W in combination with ramucirumab 8 mg/kg Q2W. Patients were treated in part B once the corresponding dose level in part A was declared safe. Part C was conducted after the recommended Phase 2 dose (RP2D) for LY3127804 was determined. In part C, LY3127804 20 mg/kg IV Q2W was administered with ramucirumab 12 mg/kg Q2W.

All doses of LY3127804 and ramucirumab were administered on days 1 and 15 of the 28-day treatment cycle. Ramucirumab was administered at least 60 min after LY3127804 dose. Total doses and planned duration of infusion for LY3127804 and ramucirumab are presented in Supplementary Table S1. Intrapatient dose-escalation was not allowed. Patients discontinued treatment due to unacceptable toxicity, progression of disease, investigator/sponsor decision or withdrawal of informed consent by the patient. Dose-limiting toxicity (DLT) period was defined as the first 28 days after first dosing (cycle 1). Dose-escalation decisions were based primarily on the safety data from the DLT period. PK data were also reviewed before dose-escalation decisions. To accommodate possible schedule conflicts, treatment was administered up to 3 days after the scheduled dosing day. No shortening of the treatment cycle was allowed.

### Objectives and assessments

The primary objective was to determine the RP2D and schedule of LY3127804 as monotherapy and combination with ramucirumab. The secondary objectives included characterisation of safety and maximum tolerated dose (MTD), PK-PD assessment of LY3127804, evaluation of limited PK (peak and trough concentrations) of ramucirumab in combination with LY3127804 and antibody formation against LY3127804. All assessments were made for LY3127804 with or without ramucirumab. Data were collected until the MTD was reached or the RP2D was determined, whichever occurred first.

Safety was assessed throughout the study using the National Cancer Institute-Common Terminology Criteria for Adverse Events (NCI-CTCAE, v4.0).^17^ A DLT was defined as any of the following adverse events (AEs) (≥grade 3 nonhematological toxicity despite maximal medical management; grade 4 haematological toxicity for > 5 days; febrile neutropenia; grade 4 thrombocytopenia or Grade 3 thrombocytopenia complicated with ≥ grade 2 bleeding; newly developed oedema characterised by a body weight increase of >10% as compared to the start of therapy; any toxicity deemed significant by investigator), possibly related to LY3127804 and occurring during the DLT period in parts A, B and C. MTD was defined as the highest tested dose in a single-agent setting that has <33% probability of causing a DLT.

PK evaluation included maximum observed drug concentration (C_max_), area under the concentration-time curve (AUC: from time zero to time t, for which t was the last time point with a measurable concentration [AUC_0-tlast_], from time zero to infinity [AUC_0-∞_], during a dosing interval at steady state [AUCτ_-ss_), volume of distribution (V_d_) and clearance (CL) for LY3127804 monotherapy and in combination with ramucirumab. We used a standard noncompartmental analysis method for PK parameter estimates for LY3127804. Pre-dose samples of LY3127804 were collected on day 1 and day 15, with post-LY3127804 dose samples collected on day 2 and day 16 of the cycle 1 and 2. For patients receiving LY3127804 + ramucirumab, sampling was done immediately after the end of ramucirumab infusion. In order to determine plasma concentration of LY3127804, 3 mL samples were collected. We used validated antigen capture enzyme-linked immunosorbent assays to determine plasma concentrations of LY3127804 and serum samples of ramucirumab. Sampling for PD/biomarker assessment followed the same schedule as for PK and samples were tested for relationship between LY3127804 concentration and biomarkers such as Ang-2/Ang-1 ratio, placenta growth factor (PLGF), VEGF (VEGFA, VEGFC and VEGFD) and soluble receptors (VEGFR2, sTie-2 and sTie-1).

The study was not designed to make an efficacy assessment; we summarised the observed tumour response data in all patients. Until week 8, radiological tumour assessment was performed at the end of each cycle (preferably between day 22 and day 29) and response to treatment was determined using RECIST (v1.1).^16^ After cycle 8, tumour assessment was done after every 2–4 cycles, as clinically indicated. Best overall response (BOR) was defined as the best response recorded from the start of the study treatment until the earliest of objective progression or start of new anticancer therapy. Disease control rate (DCR) was defined as the proportion of patients who exhibited stable disease (SD) or confirmed complete response or partial response (PR) relative to baseline.

### Statistical considerations

The estimated sample size for this study was 45–60 patients. In part A, the planned enrolment was 15–30 patients, which was estimated using a 3 + 3 dose-escalation method and incidence of DLT.^18^ In parts B and C, estimated patient enrolment was 30 patients (six patients per dose level). Demographic characteristics, patient disposition and all endpoints, except any exploratory analysis, were summarised using descriptive analysis. The analysis included all patients who had received ≥1 dose of LY3127804. All continuous variables were summarised using mean [standard deviation], median (minimum − maximum), whereas categorical variables were summarised using *n* and percentages.

## Results

### Patient disposition and baseline characteristics

Between November 2015 and November 2017, 62 patients (mean age 57.3 ± 12.1 years, 58.1% males) with advanced/metastatic solid tumours were enrolled (Table 1), into the following cohorts: part A, *n* = 20; part B, *n* = 35; part C, *n* = 7. At the cut-off date (November 23, 2017), six (9.7%) patients remained on-treatment. Reasons for discontinuation of the study treatment included progression of disease (71%), withdrawal by the patient (4.8%), AEs (3.2%), and death due to study disease (1.6%). Six patients (9.7%) were lost to follow-up.

**Table 1:** Demographics and baseline characteristics.

Parameter	Total parts A-C (*N* = 62)	Part A (*n* = 20) LY monotherapy	Part B (*n* = 35) LY + 8 mg/kg ramucirumab	Part C (*n* = 7) LY + 12 mg/kg ramucirumab
Age (years), mean^a^	57.3 ± 12.1	60.6 ± 4.6	56.2 ± 5.5	54.4 ± 9.1
Sex				
Female	26 (41.9)	11 (55.0)	12 (34.3)	3 (42.9)
Male	36 (58.1)	9 (45.0)	23 (65.7)	4 (57.1)
Age group				
<65 years	41 (66.1)	13 (65.0)	22 (62.9)	6 (85.7)
≥65 years	21 (33.9)	7 (35.0)	13 (37.1)	1 (14.3)
Race				
Black/African American	4 (6.4)	1 (5.0)	3 (8.6)	0 (0)
Caucasian	56 (90.3)	17 (85.0)	32 (91.4)	7 (100)
Missing data	2 (3.3)	2 (10.0)	0 (0)	0 (0)
Ethnicity				
Hispanic/Latino	1 (1.6)	0 (0)	1 (2.9)	0 (0)
Non-Hispanic/Latino	45 (72.6)	14 (70.0)	26 (74.3)	5 (71.4)
Not reported	16 (25.8)	6 (30.0)	8 (22.9)	2 (28.6)
Patients by country				
US	18 (29.0)	6 (30.0)	10 (28.6)	2 (28.6)
Belgium	15 (24.2)	5 (25.0)	9 (25.7)	1 (14.3)
Spain	12 (19.4)	3 (15.0)	8 (22.9)	1 (14.3)
France	17 (27.4)	6 (30.0)	8 (22.9)	3 (42.9)
Treatment status at cut-off				
On-treatment	6 (9.7)	0 (0)	3 (8.6)	3 (42.9)
Off-treatment	56 (90.3)	20 (100)	32 (91.4)	4 (57.1)

All data are reported as *n* (%), unless specified.

^a^Mean values presented with standard deviation.

For LY3127804, the median number of cycles per patient was 2 (1–9), 3 (1–19) and 4 (2–5) in parts A, B and C, respectively. The median duration of treatment in parts A, B and C was 8.6 (4–37) weeks, 11.1 (2–80) weeks and 16.7 (6–20) weeks, respectively. Supplementary Table S2 presents the drug exposure by cohorts in part A and part B.

### Safety, toxicity and RP2D

No DLT was reported in any of the cohorts. Therefore, the MTD of LY3127804 was not reached. One patient discontinued the study due to grade 3 hyperbilirubinemia (unrelated to treatment) in cohort B3 and one patient discontinued the study drug due to treatment-related grade 3 hypertension in part C.

Serious AEs (SAEs), regardless of the causality, occurred in 3, 11 and 3 patients in parts A, B and C, respectively (Table 2). Of the four patients with treatment-related SAEs, three were in part B and one was in part C. Grade ≥ 3 events of hypertension (*n* = 3, 2 in cohorts B5 and B6 and 1 in part C), abdominal pain (*n* = 1 in cohort B5) and infusion-related reaction (*n* = 1 in cohort B3) were reported as treatment-related SAEs. Grade ≥ 3 hypertension cases were managed with medical treatment and occurred outside the DLT period. The 20 mg/kg Q2W dose was determined as the RP2D for LY3127804, both as monotherapy and in combination with the two doses of ramucirumab.

**Table 2:** Summary of all-cause TEAEs and SAEs.

AEs	Part A (LY monotherapy)						
	Total Part A (*N* = 20)	Cohort A1 LY 4 mg/kg (*n* = 3)	Cohort A2 LY 8 mg/kg (*n* = 4)	Cohort A3 LY 12 mg/kg (*n* = 3)	Cohort A4 LY 16 mg/kg (*n* = 3)	Cohort A5 LY 20 mg/kg (*n* = 3)	Cohort A6 LY 27 mg/kg (*n* = 4)
TEAEs							
All grades	20 (100)	3 (100)	4 (100)	3 (100)	3 (100)	3 (100)	4 (100)
Grade 3/4	7 (35.0)	0 (0)	1 (25.0)	1 (33.3)	1 (33.3)	0 (0)	4 (100)
Grade 5	0 (0)	0 (0)	0 (0)	0 (0)	0 (0)	0 (0)	0 (0)
SAEs^a^	3 (15.0)	–	–	–	–	–	–

Part B (LY + RAM 8 mg/kg)							Part C (LY + RAM 12 mg/kg)
	Total Part B (*n* = 35)	Cohort B2 LY 8 mg/kg (*n* = 6)	Cohort B3 LY 12 mg/kg (*n* = 7)	Cohort B4 LY 16 mg/kg (*n* = 7)	Cohort B5 LY 20 mg/kg (*n* = 7)	Cohort B6 LY 27 mg/kg (*n* = 8)	LY 20 mg/kg (*n* = 7)
TEAEs							
All grades	35 (100)	6 (100)	7 (100)	7 (100)	7 (100)	8 (100)	7 (100)
Grade 3/4	20 (57.1)	2 (33.3)	5 (71.4)	5 (71.4)	2 (28.6)	6 (75.0)	6 (85.7)
Grade 5	1 (16.6)	0 (0)	0 (0)	0 (0)	1 (16.6)	0 (0)	0 (0)
SAEs^a^	11 (31.4)	–	–	–	–	–	3 (42.9)

All data are reported as *n* (%), unless specified.

*LY* LY3127804, *RAM* ramucirumab.

^a^Data not available by cohort.

Treatment-emergent adverse event (TEAE) occurred in all patients. Treatment-related AEs occurred in 41 patients (66.1%). Grade ≥ 3 TEAEs were reported in 34 patients (54.8%) (Table 2), of which 12 patients (19.30%) had treatment-related grade ≥ 3 AEs. In part A, the most frequently occurring TEAEs included constipation, diarrhoea, fatigue and peripheral oedema, occurring in 20% of patients each (Supplementary Table S3). Fatigue (10%) was the most common treatment-related AEs in part A (Table 3).

**Table 3:** Treatment-related TEAEs in ≥5% of patients—part A and part B.

TEAEs	All grade	Grade ≥ 3	All grade	Grade ≥ 3	All grade	Grade ≥ 3	All grade	Grade ≥ 3	All grade	Grade ≥ 3	All grade	Grade ≥ 3	All grade	Grade ≥ 3
TEAEs in part A	Total part A (*n* = 20)		Cohort A1 LY 4 mg/kg (*n* = 3)		Cohort A2 LY 8 mg/kg (*n* = 4)		Cohort A3 LY 12 mg/kg (*n* = 3)		Cohort A4 LY 16 mg/kg (*n* = 3)		Cohort A5 LY 20 mg/kg (*n* = 3)		Cohort A6 LY 27 mg/kg (*n* = 4)	
Fatigue	2 (10.0)	0 (0)	0 (0)	0 (0)	1 (25.0)	0 (0)	0 (0)	0 (0)	0 (0)	0 (0)	0 (0)	0 (0)	1 (25.0)	0 (0)
Constipation	1 (5.0)	0 (0)	0 (0)	0 (0)	1 (25.0)	0 (0)	0 (0)	0 (0)	0 (0)	0 (0)	0 (0)	0 (0)	0 (0)	0 (0)
Arthralgia	1 (5.0)	0 (0)	0 (0)	0 (0)	0 (0)	0 (0)	0 (0)	0 (0)	1 (33.3)	0 (0)	0 (0)	0 (0)	0 (0)	0 (0)
Diarrhoea	1 (5.0)	0 (0)	0 (0)	0 (0)	0 (0)	0 (0)	1 (33.3)	0 (0)	0 (0)	0 (0)	0 (0)	0 (0)	0 (0)	0 (0)
Dizziness	1 (5.0)	0 (0)	0 (0)	0 (0)	1 (25.0)	0 (0)	0 (0)	0 (0)	0 (0)	0 (0)	0 (0)	0 (0)	0 (0)	0 (0)
Peripheral oedema	1 (5.0)	0 (0)	0 (0)	0 (0)	0 (0)	0 (0)	0 (0)	0 (0)	0 (0)	0 (0)	0 (0)	0 (0)	1 (25.0)	0 (0)
Dysgeusia	1 (5.0)	0 (0)	0 (0)	0 (0)	1 (25.0)	0 (0)	0 (0)	0 (0)	0 (0)	0 (0)	0 (0)	0 (0)	0 (0)	0 (0)
Headache	1 (5.0)	0 (0)	0 (0)	0 (0)	0 (0)	0 (0)	0 (0)	0 (0)	0 (0)	0 (0)	0 (0)	0 (0)	1 (25.0)	0 (0)
Hypertension	1 (5.0)	1 (5.0)	0 (0)	0 (0)	0 (0)	0 (0)	0 (0)	0 (0)	0 (0)	0 (0)	0 (0)	0 (0)	1 (25.0)	1 (25.0)
Malaise	1 (5.0)	0 (0)	0 (0)	0 (0)	0 (0)	0 (0)	0 (0)	0 (0)	0 (0)	0 (0)	0 (0)	0 (0)	1 (25.0)	0 (0)
Proteinuria	1 (5.0)	0 (0)	0 (0)	0 (0)	1 (25.0)	0 (0)	0 (0)	0 (0)	0 (0)	0 (0)	0 (0)	0 (0)	0 (0)	0 (0)
Weight increase	1 (5.0)	0 (0)	1 (33.3)	0 (0)	0 (0)	0 (0)	0 (0)	0 (0)	0 (0)	0 (0)	0 (0)	0 (0)	0 (0)	0 (0)

TEAEs in part B	Total part B LY + RAM 8 mg/kg (*N* = 35)		–		Cohort B2 LY 8 mg/kg + (*n* = 6)		Cohort B3 LY 12 mg/kg (*n* = 7)		Cohort B4 LY 16 mg/kg (*n* = 7)		Cohort B5 LY 20 mg/kg (*n* = 7)		Cohort B6 LY 27 mg/kg (*n* = 8)	
Hypertension	12 (34.3)	6 (17.1)	–	–	2 (33.3)	1 (16.7)	1 (14.3)	1 (14.3)	2 (28.6)	0 (0)	3 (42.9)	2 (28.6)	4 (50.0)	2 (25.0)
Fatigue	8 (22.9)	0 (0)	–	–	3 (50.0)	0 (0)	0 (0)	0 (0)	2 (28.6)	0 (0)	1 (14.3)	0 (0)	2 (25.0)	0 (0)
Peripheral oedema	7 (20.0)	0 (0)	–	–	1 (16.7)	0 (0)	0 (0)	0 (0)	2 (28.6)	0 (0)	2 (28.6)	0 (0)	2 (25.0)	0 (0)
Diarrhoea	4 (11.4)	0 (0)	–	–	1 (16.7)	0 (0)	1 (14.3)	0 (0)	1 (14.3)	0 (0)	1 (14.3)	0 (0)	0 (0)	0 (0)
Infusion-related reaction	4 (11.4)	1 (2.9)	–	–	0 (0)	0 (0)	1 (14.3)	1 (14.3)	1 (14.3)	0 (0)	0 (0)	0 (0)	2 (25.0)	0 (0)
Vomiting	4 (11.4)	1 (2.9)	–	–	0 (0)	0 (0)	0 (0)	0 (0)	1 (14.3)	0 (0)	0 (0)	0 (0)	3 (37.5)	1 (12.5)
Headache	3 (8.6)	0 (0)	–	–	1 (16.7)	0 (0)	0 (0)	0 (0)	1 (14.3)	0 (0)	0 (0)	0 (0)	1 (12.5)	0 (0)
Abdominal pain	2 (5.7)	0 (0)	–	–	0 (0)	0 (0)	1 (14.3)	0 (0)	0 (0)	0 (0)	1 (14.3)	0 (0)	0 (0)	0 (0)
Arthralgia	2 (5.7)	0 (0)	–	–	0 (0)	0 (0)	0 (0)	0 (0)	1 (14.3)	0 (0)	0 (0)	0 (0)	1 (12.5)	0 (0)
Asthenia	2 (5.7)	0 (0)	–	–	0 (0)	0 (0)	0 (0)	0 (0)	1 (14.3)	0 (0)	0 (0)	0 (0)	1 (12.5)	0 (0)
Decreased appetite	2 (5.7)	0 (0)	–	–	1 (16.7)	0 (0)	0 (0)	0 (0)	0 (0)	0 (0)	0 (0)	0 (0)	1 (12.5)	0 (0)

All data are reported as *n* (%), unless specified.

*LY* LY3127804, *RAM* ramucirumab.

In part B, hypertension and peripheral oedema were the most common TEAEs (42.9% each) followed by fatigue (28.6%), headache (25.7%) and vomiting (22.9%; Supplementary Table S4). The most frequent treatment-related AEs in part B were hypertension (34.3%), fatigue (22.9%) and peripheral oedema (20.0%) (Table 3). Hypertension (57.1%) and constipation (42.9%) were the most common TEAEs in part C, with 42.9% of patients having study treatment-related hypertension. Dose-modifications were made in 13 patients overall (21%); four in part A and nine in part B.

Twelve deaths (19.4%) were reported during the study, four in part A (one each in A3 and A5, and two in A6) and eight in part B (one in B3, two each in B4 and B5 and three in B6). Progressive disease caused nine deaths, six during treatment and three after 30 days of discontinuing study treatment. One death due to a TEAE of pharyngeal haemorrhage occurred during treatment in cohort B5 (LY3127804 20 mg/kg + ramucirumab 8 mg/kg). The patient had received high dose radiotherapy to the bleeding area. The event was not considered unequivocally related to study treatment. Two patients died due to an unknown cause 30 days after discontinuing study treatment.

### PK-PD evaluation

**Fig. 1:**
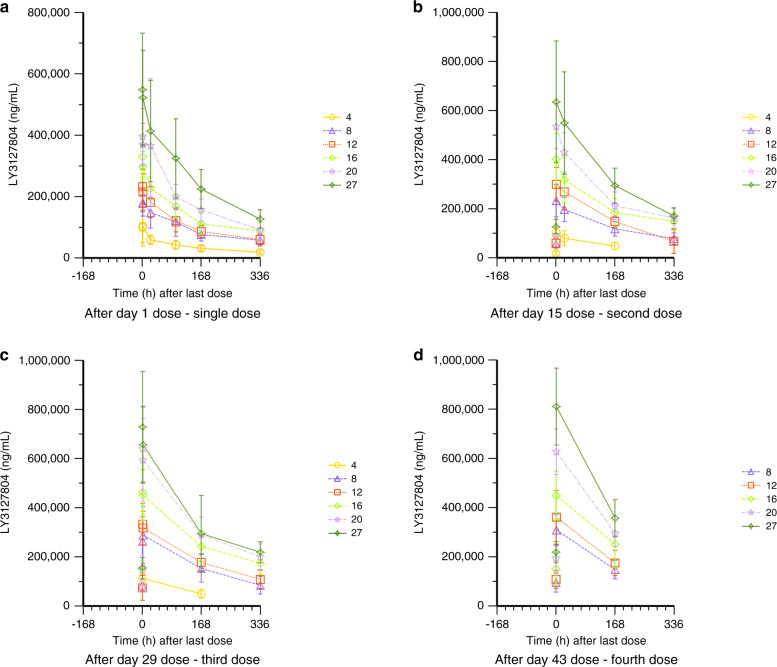
Mean (±SD) LY3127804 concentration versus time profile. The legend indicates LY3127804 dose level in mg/kg.

**Fig. 2:**
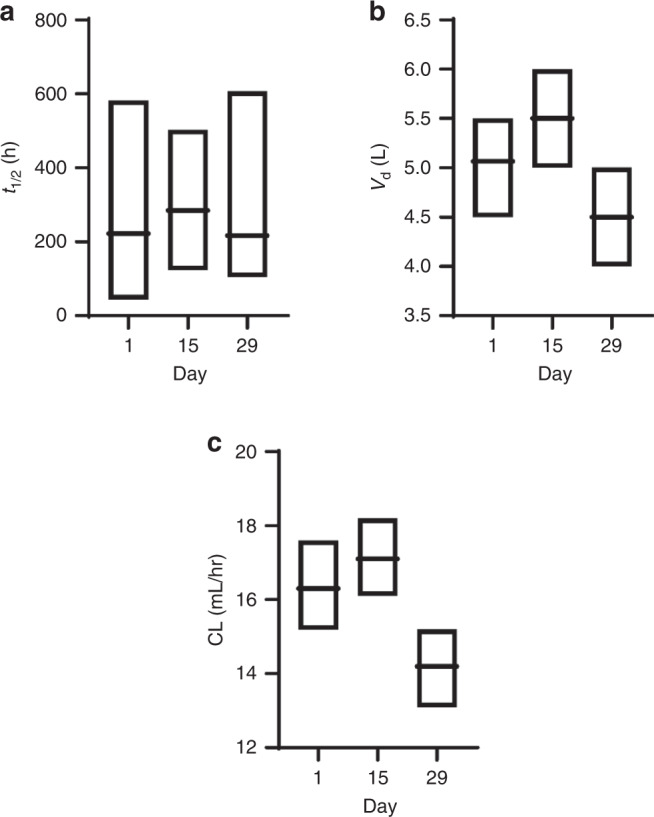
Geometric mean (and range) of t_1/2_, V_d_ and CL for LY3127804. All data presented as mean (SD). The parameters are presented after single administration (day 1*, n* = 56), two doses (day 15, *n* = 49) and three doses (day 29, *n* = 37*)*.

Mean plasma concentration of LY3127804 after single or multiple doses increased with higher doses (Fig. 1). LY3127804 CL was similar following administration as single agent and in combination with ramucirumab. The combined PK data from all parts showed a constant CL and terminal half-life (t_1/2_) for LY3127804, irrespective of the dose. Consequently, AUC_(0-336)_ increased in a dose-proportional manner for each dose and each day of dosing (Table 4). Mean CL, V_d_ and t_1/2_ for LY3127804 across the study were 16.3 mL/h, 5.2 L and 222 h, respectively. The CL, V_d_ and t_1/2_ of LY3127804 at day 1, day 15 and day 29 are presented in Fig. 2.

**Table 4:** PK assessment with all LY3127804 doses at different administration times.

LY dose (mg/kg)	Day	*N*	C_max_ (ng/mL)	AUC_(0-336)_ (µg·h/mL)	AUC_(0-∞)_ (µg·h/mL)	CL (mL/h)	t_1/2_ (h)
4	1	3	96,867 (57)	11,631 (30)	16,737 (34)	16.5 (28)	199 (16)
	15	3	100,120 (22)	16,647 (33)	–	16.8 (25)	202 (21)
	29	3/2^a^	109,960 (27)	16,136 (27)^a^	–	15.3 (31)^a^	211 (27)^a^
	43	2	96,380 (26)	–	–	–	–
8	1	9/6^a^	187,210 (20)	29,817 (25)^a^	44,854 (31)	15.3 (13)^a^	202 (21)^a^
	15	9	225,140 (28)	42,486 (22)	–	14.4 (22)	240 (36)
	29	6/5^a^	310,580 (36)	53,889 (41)^a^	–	12.2 (40)^a^	211 (55)^a^
	43	9	303,320 (21)	–	–	–	–
12	1	9	231,190 (29)	33,036 (31)	50,661 (40)	18.5 (43)	220 (33)
	15	8/6^a^	294,290 (32)	47,084 (35)	–	20.3 (42)	199 (25)^a^
	29	9/8^a^	342,140 (24)	62,327 (26)	–	15.5 (35)	209 (24)^a^
	43	8	390,170 (13)	–	–	–	–
16	1	9	313,230 (22)	43,636 (25)	73,477 (46)	15.5 (38)	255 (41)
	15	10	347,080 (39)	65,837 (39)	–	16.8 (24)	270 (26)
	29	8/5^a^	480,120 (23)	93,032 (25)	–	13.0 (26)	248 (7.1)^a^
	43	9	439,330 (22)	–	–	–	–
20	1	17	423,120 (35)	57,917 (23)	91,311 (32)	15.8 (33)	228 (50)
	15	13	531,190 (17)	85,529 (22)	–	16.9 (25)	227 (25)
	29	14/10^a^	631,940 (27)	106,655 (28)	–	13.4 (25)	238 (38)^a^
	43	10	620,870 (16)	–	–	–	–
27	1	12	551,370 (36)	81,931 (28)	122,518 (34)	16.6 (38)	211 (39)
	15	9	608,330 (38)	108,015 (24)	–	18.2 (21)	202 (42)
	29	10/8^a^	721,420 (30)	120,607 (29)^a^	–	15.9 (23)^a^	185 (19)^a^
	43	8	797,710 (19)	–	–	–	–

All data reported as geometric mean (coefficient of variation %), unless specified.

As CL of LY3127804 monotherapy and LY3127804 combination is similar, combined PK data are reported for parts A, B, and C.

^a^N as (X1/X2*): C_max_ was determined in X1 patients and t_1/2_ (hence AUC_(0-∞)_ and CL) could only be determined in X2 patients.

Ang-2 concentrations increased substantially with LY3127804 therapy, possibly due to the binding of LY3127804 to Ang-2 leading to decrease of Ang-2 clearance or turnover rate. Similarly, an increase in Ang-1 levels was observed under LY3127804 treatment. PLGF concentration increased substantially in the combination arm (part B and C) as has been shown previously with ramucirumab treatment alone, while VEGFC increased moderately with LY3127804 and ramucirumab combination treatments (Supplementary Figs. S2 and S3). The postbaseline, on-treatment VEGFD data are very limited and suggest a moderate increase under treatment, but that trend is within the substantial interpatient CV.

### Immunogenicity

We collected 371 samples for immunogenicity analysis, of which 332 (90%) were analysed. Of the 59 patients with evaluable data, treatment-emergent antidrug antibodies (TE ADA) were detected in six patients (10.2%). There was no correlation between the LY3127804 dose and TE ADA detection. Titre range for TE ADA was 1:10–1:80.

### Efficacy

Confirmed PR as BOR was achieved by four patients: two patients from cohort B2 (one each with clear cell endometrial carcinoma and cervix squamous cell carcinoma), one patient from cohort B6 (carcinoma of unknown primary) and one patient from part C (gastroesophageal junction adenocarcinoma). Eleven of 20 patients (55.0%) in part A, 13 of 35 patients (37.1%) in part B and five of seven patients (71.4%) in part C achieved SD; whereas 8 (40.0%), 15 (42.9%), and 1 (14.3%) had progressive disease in parts A, B and C, respectively. Three patients were not evaluable for efficacy. Among the tumour types, seven patients with colon carcinoma achieved SD (one each in cohorts A1, A2, A3, A4, B2, B3 and part C), with duration of SD presented in Supplementary Fig. S4. Other tumour types with BOR and the mean duration of treatment in patients in parts A, B and C are presented in Supplementary Fig. S4. BOR was not evaluable in one patient each from part A and part B. Overall response rate (ORR) was zero in part A, 8.6% in part B and 14.3% in part C. DCR was 55.0%, 45.7% and 85.7% in parts A, B and C, respectively.

## Discussion

This is the first study to assess the novel anti-Ang-2 MAb LY3127804 as monotherapy and in combination with ramucirumab in patients with advanced solid tumours. The study showed a favourable safety profile for both LY3127804 alone and in combination with ramucirumab. As no DLT occurred and the MTD was not reached, LY3127804 20 mg/kg was defined as the RP2D. The LY3127804 PK, being linear, did not support determination of a target-mediated drug disposition PK model. Consequently, LY3127804 RP2D was determined based on the observed safety/tolerability profile, the on-target effects of hypertension and oedema indicated that the relevant biological dose had been reached. In addition, objective responses were observed at doses below 27 mg/kg. Consequently 20 mg/kg was considered a reasonable RP2D, both for monotherapy and combination.

In our study, fatigue and constipation were the most common treatment-related AEs with LY3127804 monotherapy. Hypertension, constipation, peripheral oedema, fatigue and abdominal pain were the main treatment-related AEs with the LY3127804 and ramucirumab combination. In addition, more than half of the patients treated with LY3127804 monotherapy achieved SD, whereas four patients treated with the LY3127804 and ramucirumab combination showed PR. The findings for safety and tolerability in our study were consistent with the findings for other Ang-2 inhibitors. Phase 1 studies of trebananib (AMG 386; first Ang-1 and Ang-2 blocker) also showed acceptable toxicity profile in patients with solid tumours.^5,19,20^ Fatigue, abdominal pain and peripheral oedema were the main TEAEs,^5^ as with LY3127804. Trebananib doses up to 15 mg/kg with chemotherapy and up to 30 mg/kg as monotherapy were considered safe in adults.^19,20^ RP2D of trebananib was 30 mg/kg in paediatric patients with solid tumours.^21^ The RP2D of nesvacumab was 20 mg/kg, with a TEAE profile similar to that of LY3127804.^9^ The MTD was not reached with all doses of MEDI3617 monotherapy and its combinations.^8^ AMG 780 (an Ang-1 and Ang-2 inhibitor),^22^ and vanucizumab (a bispecific anti-Ang-2/VEGF MAb) were considered safe in patients with advanced solid tumours.^23^

In this Phase 1 study, LY3127804 monotherapy and its combination with ramucirumab demonstrated dose-dependent-proportional increase in LY3127804 concentration and AUC. This finding characterised LY3127804 pharmacokinetics as being linear in the dose range studied with a constant CL and t_1/2_. LY3127804 PK properties are consistent with reported PK properties for large MAb molecules. These observations were consistent with findings from prior PK studies evaluating the C_max_, AUC, CL for trebananib monotherapy,^20^ and the combination of trebananib with chemotherapy^19^ and in combination with sorafenib and sunitinib.^6^

Response assessments with LY3127804 monotherapy (SD: 55%; PR: 0%) were consistent with those reported with trebananib in patients with advanced solid tumours (SD: 55%%; PR: 3.5%)^5^ and nesvacumab (SD: 48.9%; PR: 2.3%).^9^ Results from the small cohorts of patients receiving various doses of the combination of LY3127804 with ramucirumab suggest a similar SD (42.8%) but with a higher proportion of patients with PR (9.5%). Therefore, LY3127804 as monotherapy and in combination with ramucirumab showed signs of clinical activity.

The future clinical development of Ang-2 inhibitors such as LY3127804 is likely to be in combination with other anti-angiogenic drugs or with compounds with different mechanisms of action, given their favourable toxicity profile in combinatory regimes. Indeed, combination of anti-angiogenic drugs with immune checkpoint inhibitors (ICIs) has already yielded good results in some advanced solid tumours.^24^ The ICIs reactivate the exhausted T-cell-mediated immune response, which is suppressed by different mechanisms including angiogenesis.^25,26^ A number of recent trials have demonstrated the efficacy of ICI and anti-angiogenic combinations in patients with different tumour types. Thus, the Phase 1b study of pembrolizumab and axitinib combination treatment in patients with advanced renal cell carcinoma showed a good safety profile and signs of clinical activity.^27^ The subsequent Phase 3 trial confirmed superiority of the combinatory treatment over sunitinib monotherapy in ORR, progression-free survival (PFS) and overall survival (OS).^28^ Similar benefits in PFS were observed with the combination of avelumab plus axitinib^29^ and atezolizumab plus bevacizumab.^30^

Other combinations, for instance pembrolizumab plus ramucirumab, have been reported to have manageable safety profile and signs of activity in other tumour types such as biliary tract cancer, NSCLC, gastroesophageal cancer, or urothelial carcinomas.^31,32^ Inhibition of angiogenesis-mediated immune suppression with the anti-Ang-2 therapy trebananib is under exploration in combination with pembrolizumab.^33^ Based on the available evidence, the effect of LY3127804 combined with ICIs should be explored in further studies.

In summary, this first-in-human, dose-escalation, Phase 1 study reported a favourable safety profile for LY3127804 with 20 mg/kg Q2W dose determined as the RP2D for LY3127804 in patients with advanced solid tumours. Furthermore, achievement of SD with LY3127804 monotherapy and PR with LY3127804 and ramucirumab combination indicates signs of clinical activity.

## Supplementary information

Supplementary Tables and Figures

## Data Availability

Lilly provides access to all individual participant data collected during the trial, after anonymisation, with the exception of pharmacokinetic or genetic data. Data are available to request 6 months after the indication studied has been approved in the US and EU and after primary publication acceptance, whichever is later. No expiration date of data requests is currently set once they are made available. Access is provided after a proposal has been approved by an independent review committee identified for this purpose and after receipt of a signed data-sharing agreement. Data and documents, including the study protocol, statistical analysis plan, clinical study report and blank or annotated case report forms, will be provided in a secure data-sharing environment for up to 2 years per proposal. For details on submitting a request, see the instructions provided at www.clinicalstudydatarequest.com.
