# Supplementary material for: First-in-human, dose-escalation, phase 1 study of anti-angiopoietin-2 LY3127804 as monotherapy and in combination with ramucirumab in patients with advanced solid tumours
Source: Br J Cancer. 2020 Aug 3;123(8):1235–43. doi: 10.1038/s41416-020-1011-7 (PMC7555513; doi:10.1038/s41416-020-1011-7)
Supplement: Supplementary file 1 — Supplementary Tables and Figures [file 41416_2020_1011_MOESM1_ESM.docx]

**Table S1. Total dose and dose duration for LY3127804 and ramucirumab**

| **Dose** | **Total Dose** | **Infusion Duration (mins), mean ± SD** |
| --- | --- | --- |
| LY3127804 – 4, 8, and 12 mg/kg | ≤1500 mg | 60 ± 10 |
|  | >1500 - 2250 mg | 90 ± 15 |
|  | >2250 mg | 120 ± 20 |
| LY3127804 – 16 and 20 mg/kg | ≤2250 mg | 90 ± 15 |
|  | >2250 mg | 120 ± 20 |
| Ramucirumab, 8 mg/kg or 12 mg/kg | - | 60 ± 10 |

**Table S2. Exposure to treatment drugs at each dose level**

| **LY3127804 Exposure** | **Cohort** | | | | | |
| --- | --- | --- | --- | --- | --- | --- |
| **Part A** | **A1 (n = 3) LY 4 mg/kg** | **A2 (n = 4) LY 8 mg/kg** | **A3 (n = 3) LY 12 mg/kg** | **A4 (n = 3) LY 16 mg/kg** | **A5 (n = 3) LY 20 mg/kg** | **A6 (n = 4) LY 27 mg/kg** |
| No. of cycles/patient | 4 (4–4) | 3.5 (2-6) | 2 (2-3) | 2 (2-3) | 1 (1-2) | 2 (2-9) |
| Therapy duration (weeks) | 16.1 (16-16) | 14.4 (8-25) | 8 (8-12) | 8.3 (8-12) | 4 (4-8) | 8.5 (6-37) |
| Cumulative dose (mg) | 2032  (1936-2824) | 4112  (1528-9024) | 4224  (2860-6120) | 5120  (4736-8016) | 2240  (1780-4776) | 8370  (4779-32640) |
| **Part B** | - | **B2 (n = 6) LY 8 mg/kg + RAM 8 mg/kg** | **B3 (n = 7) LY 12 mg/kg + RAM 8 mg/kg** | **B4 (n = 7) LY 16 mg/kg + RAM 8 mg/kg** | **B5 (n = 7) LY 20 mg/kg + RAM 8 mg/kg** | **B6 (n = 8) LY 27 mg/kg + RAM 8 mg/kg** |
| No. of cycles/patient | - | 4 (2-19) | 2 (1-8) | 4 (2-13) | 3 (2-14) | 2 (1-9) |
| Therapy duration (weeks) | - | 16.5 (8-80) | 8.4 (2-33) | 16.3 (8-51) | 10.1 (6-60) | 8.1 (2-36) |
| Cumulative dose (mg) | - | 5468  (2720-21600) | 5184  (792-14544) | 7641.6  (3728-34522) | 5964  (4210-56836) | 7706  (2009-31671) |

*Abbreviation: LY=LY3127804; RAM=ramucirumab.*

*All data are reported as median (range), unless specified.*

**Table S3. All cause TEAEs in ≥10% patients – Part A (total and by cohort)**

| **TEAE** | **Total Part A (N = 20)** | | **Cohort A1  LY 4 mg/kg  (n = 3)** | | **Cohort A2  LY 8 mg/kg  (n = 4)** | | **Cohort A3  LY 12 mg/kg  (n = 3)** | | **Cohort A4 LY 16 mg/kg  (n = 3)** | | **Cohort A5 LY 20 mg/kg  (n = 3)** | | **Cohort A6  LY 27mg/kg  (n = 4)** | |
| --- | --- | --- | --- | --- | --- | --- | --- | --- | --- | --- | --- | --- | --- | --- |
|  | **All Grade** | **Grade** **≥3** | **All Grade** | **Grade ≥3** | **All Grade** | **Grade ≥3** | **All Grade** | **Grade ≥3** | **All Grade** | **Grade ≥3** | **All Grade** | **Grade ≥3** | **All Grade** | **Grade ≥3** |
| Constipation | 4 (20.0) | 0 (0) | 0 (0) | 0 (0) | 1 (25.0) | 0 (0) | 2 (66.7) | 0 (0) | 0 (0) | 0 (0) | 1 (33.3) | 0 (0) | 0 (0) | 0 (0) |
| Diarrhea | 4 (20.0) | 0 (0) | 1 (33.3) | 0 (0) | 1 (25.0) | 0 (0) | 1 (33.3) | 0 (0) | 0 (0) | 0 (0) | 1 (33.3) | 0 (0) | 0 (0) | 0 (0) |
| Fatigue | 4 (20.0) | 1 (5.0) | 0 (0) | 0 (0) | 1 (25.0) | 0 (0) | 0 (0) | 0 (0) | 0 (0) | 0 (0) | 0 (0) | 0 (0) | 3 (75.0) | 1 (25.0) |
| Peripheral edema | 4 (20.0) | 0 (0) | 1 (33.3) | 0 (0) | 0 (0) | 0 (0) | 0 (0) | 0 (0) | 0 (0) | 0 (0) | 1 (33.3) | 0 (0) | 2 (50.0) | 0 (0) |
| Abdominal pain | 3 (15.0) | 0 (0) | 0 (0) | 0 (0) | 2 (50.0) | 0 (0) | 1 (33.3) | 0 (0) | 0 (0) | 0 (0) | 0 (0) | 0 (0) | 0 (0) | 0 (0) |
| Headache | 3 (15.0) | 0 (0) | 0 (0) | 0 (0) | 2 (50.0) | 0 (0) | 0 (0) | 0 (0) | 0 (0) | 0 (0) | 0 (0) | 0 (0) | 1 (25.0) | 0 (0) |
| Arthralgia | 2 (10.0) | 0 (0) | 0 (0) | 0 (0) | 0 (0) | 0 (0) | 1 (33.3) | 0 (0) | 1 (33.3) | 0 (0) | 0 (0) | 0 (0) | 0 (0) | 0 (0) |
| Asthenia | 2 (10.0) | 1 (5.0) | 0 (0) | 0 (0) | 0 (0) | 0 (0) | 0 (0) | 0 (0) | 0 (0) | 0 (0) | 1 (33.3) | 0 (0) | 1 (25.0) | 1 (25.0) |
| Cough | 2 (10.0) | 0 (0) | 0 (0) | 0 (0) | 0 (0) | 0 (0) | 1 (33.3) | 0 (0) | 0 (0) | 0 (0) | 0 (0) | 0 (0) | 1 (25.0) | 0 (0) |
| Dizziness | 2 (10.0) | 0 (0) | 0 (0) | 0 (0) | 1 (25.0) | 0 (0) | 0 (0) | 0 (0) | 0 (0) | 0 (0) | 0 (0) | 0 (0) | 1 (25.0) | 0 (0) |

*Abbreviation: LY=LY3127804.*

*All data are reported as n (%), unless specified.*

**Table S4. All-cause TEAEs in ≥10% patients – Part B (LY3127804 + RAM 8 mg/kg)**

| **TEAE** | **Total Part B (N = 35)** | | **Cohort B2  LY 8 mg/kg  (n = 6)** | | **Cohort B3  LY 12 mg/kg  (n = 7)** | | **Cohort B4  LY 16 mg/kg  (n = 7)** | | **Cohort B5  LY 20 mg/kg  (n = 7)** | | **Cohort B6  LY 27 mg/kg  (n = 8)** | |
| --- | --- | --- | --- | --- | --- | --- | --- | --- | --- | --- | --- | --- |
|  | **All Grade** | **Grade ≥3** | **All Grade** | **Grade ≥3** | **All Grade** | **Grade ≥3** | **All Grade** | **Grade ≥3** | **All Grade** | **Grade ≥3** | **All Grade** | **Grade ≥3** |
| Hypertension | 15 (42.9) | 9 (25.7) | 3 (50.0) | 1 (16.7) | 2 (28.6) | 2 (28.6) | 2 (28.6) | 0 (0) | 4 (57.1) | 3 (42.9) | 4 (50.0) | 3 (37.5) |
| Peripheral edema | 15 (42.9) | 0 (0) | 2 (33.3) | 0 (0) | 2 (28.6) | 0 (0) | 3 (42.9) | 0 (0) | 4 (57.1) | 0 (0) | 4 (50.0) | 0 (0) |
| Fatigue | 10 (28.6) | 0 (0) | 3 (50.0) | 0 (0) | 0 (0) | 0 (0) | 2 (28.6) | 0 (0) | 1 (14.3) | 0 (0) | 4 (50.0) | 0 (0) |
| Headache | 9 (25.7) | 0 (0) | 3 (50.0) | 0 (0) | 1 (14.3) | 0 (0) | 3 (42.9) | 0 (0) | 1 (14.3) | 0 (0) | 1 (12.5) | 0 (0) |
| Vomiting | 8 (22.9) | 1 (2.9) | 1 (16.7) | 0 (0) | 0 (0) | 0 (0) | 1 (14.3) | 0 (0) | 0 (0) | 0 (0) | 6 (75.0) | 1 (12.5) |
| Abdominal pain | 6 (17.1) | 0 (0) | 1 (16.7) | 0 (0) | 1 (14.3) | 0 (0) | 1 (14.3) | 0 (0) | 1 (14.3) | 0 (0) | 2 (25.0) | 0 (0) |
| Constipation | 6 (17.1) | 0 (0) | 1 (16.7) | 0 (0) | 1 (14.3) | 0 (0) | 1 (14.3) | 0 (0) | 2 (28.6) | 0 (0) | 1 (12.5) | 0 (0) |
| Decreased appetite | 6 (17.1) | 0 (0) | 1 (16.7) | 0 (0) | 0 (0) | 0 (0) | 2 (28.6) | 0 (0) | 2 (28.6) | 0 (0) | 1 (12.5) | 0 (0) |
| Dyspnea | 6 (17.1) | 1 (2.9) | 0 (0) | 0 (0) | 2 (28.6) | 1 (14.3) | 2 (28.6) | 0 (0) | 1 (14.3) | 0 (0) | 1 (12.5) | 0 (0) |
| Ascites | 5 (14.3) | 1 (2.9) | 1 (16.7) | 0 (0) | 0 (0) | 0 (0) | 0 (0) | 0 (0) | 1 (14.3) | 0 (0) | 3 (37.5) | 1 (12.5) |
| Diarrhea | 5 (14.3) | 0 (0) | 1 (16.7) | 0 (0) | 1 (14.3) | 0 (0) | 1 (14.3) | 0 (0) | 1 (14.3) | 0 (0) | 1 (12.5) | 0 (0) |
| Abdominal distension | 4 (11.4) | 0 (0) | 0 (0) | 0 (0) | 0 (0) | 0 (0) | 1 (14.3) | 0 (0) | 0 (0) | 0 (0) | 3 (37.5) | 0 (0) |
| Arthralgia | 4 (11.4) | 0 (0) | 0 (0) | 0 (0) | 1 (14.3) | 0 (0) | 2 (28.6) | 0 (0) | 0 (0) | 0 (0) | 1 (12.5) | 0 (0) |
| Asthenia | 4 (11.4) | 0 (0) | 1 (16.7) | 0 (0) | 0 (0) | 0 (0) | 2 (28.6) | 0 (0) | 0 (0) | 0 (0) | 1 (12.5) | 0 (0) |
| Cough | 4 (11.4) | 0 (0) | 0 (0) | 0 (0) | 1 (14.3) | 0 (0) | 2 (28.6) | 0 (0) | 1 (14.3) | 0 (0) | 0 (0) | 0 (0) |
| Infusion-related reaction | 4 (11.4) | 1 (2.9) | 0 (0) | 0 (0) | 1 (14.3) | 1 (14.3) | 1 (14.3) | 0 (0) | 0 (0) | 0 (0) | 2 (25.0) | 0 (0) |
| Proteinuria | 4 (11.4) | 0 (0) | 0 (0) | 0 (0) | 1 (14.3) | 0 (0) | 1 (14.3) | 0 (0) | 1 (14.3) | 0 (0) | 1 (12.5) | 0 (0) |

*Abbreviation: LY=LY3127804; RAM=ramucirumab.*

*All data are reported as n (%), unless specified.*

**Table S5. Institutional review boards**

| **Site Loc Address Type** | **Site Loc Center Name** | **Country Code** | **Site Ref Number** | **Primary Investigator** |
| --- | --- | --- | --- | --- |
| ERB - Ethics Commitee Jules Bordet | Institut Jules Bordet, Brussels, Belgium | BE | 301 | Ahmad Awada |
| ERB | Hospital Universitari Vall d'Hebron, Barcelona, Spain | ES | 501 | Juan J. Martin Liberal |
| ERB - CPP "Ile-de-France V" | Gustave Roussy, Paris, France | FR | 401 | Antoine Hollebecque |
| ERB - IntegReview Ethical Review Board | Sarah Cannon Research Institute, Nashville, Tennessee, USA | US | 101 | Johanna Bendell |

**Figure S1. Study design and patient disposition**

**Total Estimated Sample size, N = 45-60**

**Part B**

**Combination Dose Exploration,**

**6 pts/cohort, *All Comer”,**

**n ~ 30 pts**

**RAM 8 mg/kg Q2W + LY3127804 8-27 mg/kg Q2W**

**Part C**

**Combination Dose Exploration,**

**6 pts/cohort, *All Comer”,**

**n ~ 6 pts**

**RAM 12 mg/kg Q2W + LY3127804 20 mg/kg Q2W**

**Part A**

**Monotherapy Dose Escalation,**

**3+3, *All Comer”,
n~15-30
LY3127804 Q2W**

**4-27 mg/kg**

*All comers are patients with any type of solid tumor.*

*Abbreviations: Q2W=once every 2 weeks; RAM=ramucirumab.*

**Figure S2. Change in Ang-1 and Ang-2 concentrations over time.**


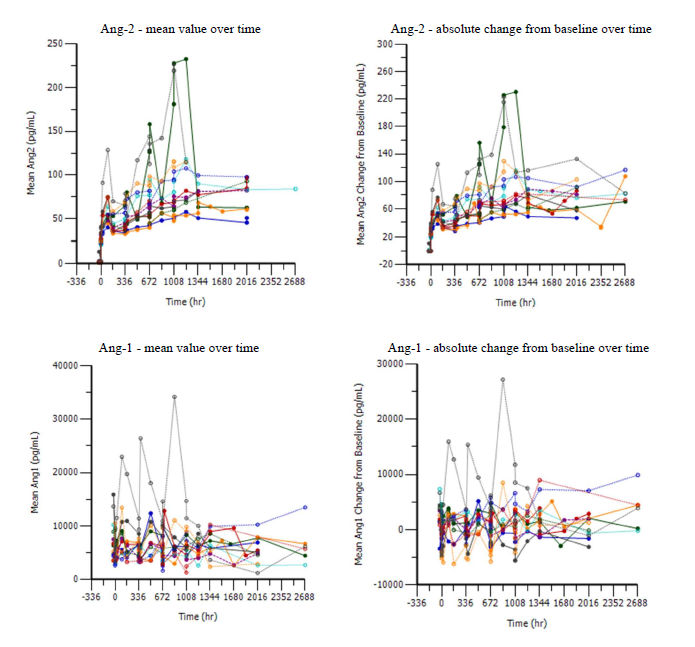


***LY3127804 monotherapy LY3127804 and ramucirumab combination***


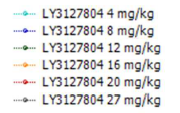

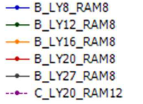


**Figure S3.** **Change in PLGF and VEGFC concentrations over time**


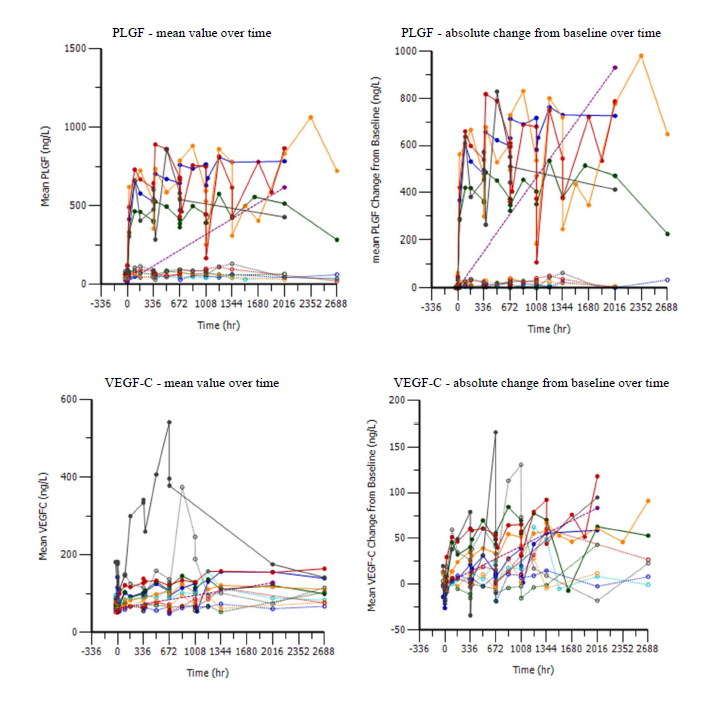


***LY3127804 monotherapy LY3127804 and ramucirumab combination***


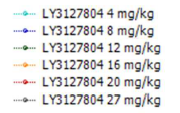

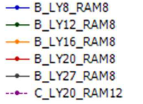


**Figure S4. Duration of treatment by tumor type in patients with PR, SD, and PD**

**
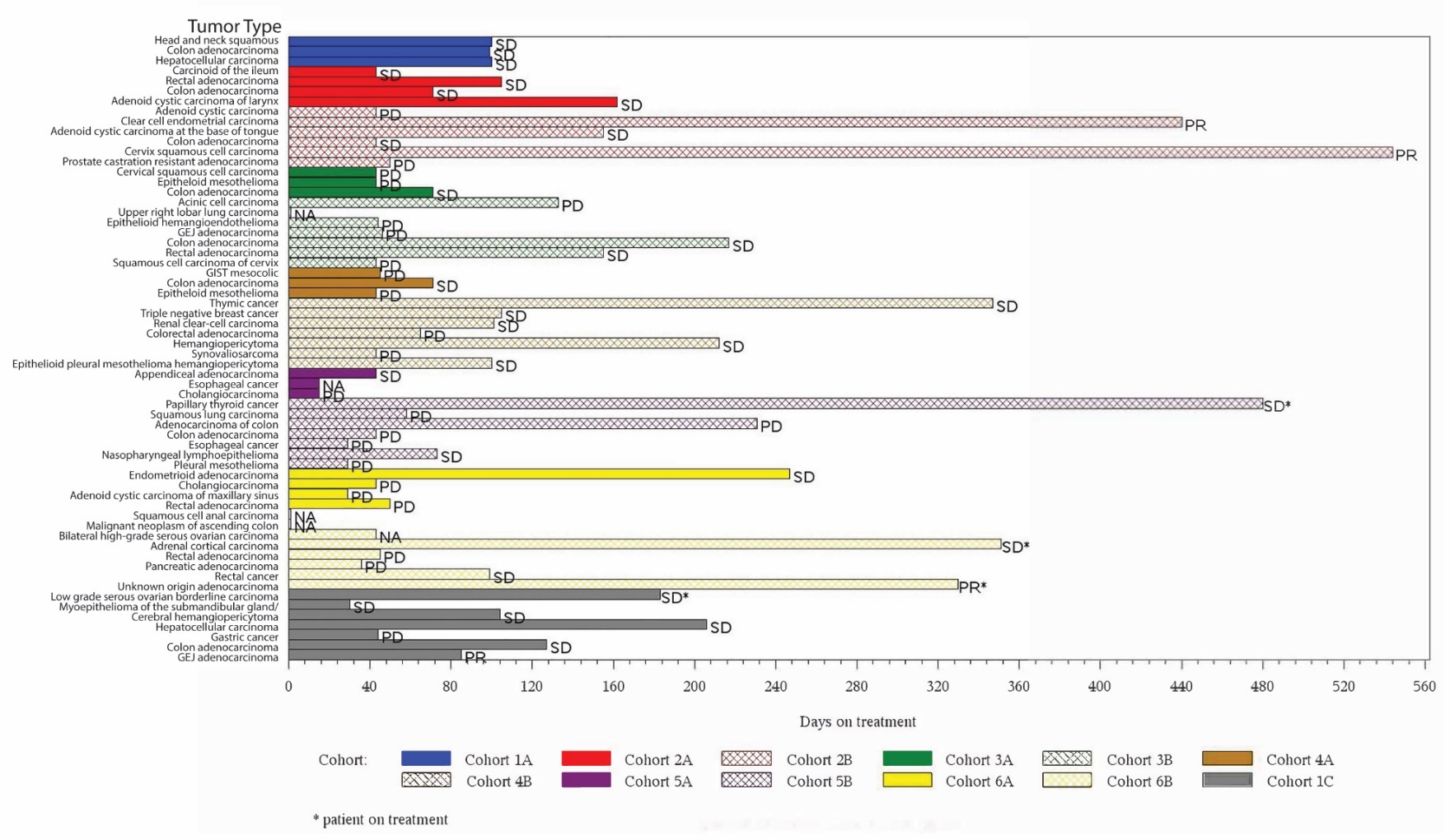
**

*Abbreviations:* *GEJ = gastroesophageal junction adenocarcinoma; GIST = gastrointestinal stromal tumor; NA = not assessed.*
